# Balancing the pendulum- the risk of universal fluid restriction and overreliance on the 'static' phase model. The Authors’ reply

**DOI:** 10.1016/j.aicoj.2026.100188

**Published:** 2026-10-14

**Authors:** Niels Van Regenmortel, Martin Vanden Eede

**Affiliations:** a Ziekenhuis aan de Stroom Campus Cadix, Department of Intensive Care Medicine Kempenstraat 100, 2030 Antwerp, Belgium; b Antwerp University Hospital, Department of Intensive Care Medicine, Drie Eikenstraat 655, 2650 Edegem, Belgium; c Ziekenhuis aan de Stroom Campus Middelheim, Department of Emergency Medicine Lindendreef 1, 2020 Antwerpen, Belgium

We thank Ghatak and Swarna for their thoughtful comments. Reassuringly, we see little disagreement with the principles outlined in our review and share their concern that the pendulum should not swing from indiscriminate fluid administration to equally indiscriminate restriction. We welcome the opportunity to clarify aspects briefly addressed in our concise review.( [1])

The authors rightly highlight the difficulty of identifying transitions between the phases of the ROSE framework. [2] We would argue that these should not be interpreted as discrete therapeutic stages with rigid boundaries, but rather as a means of continuously reassessing competing clinical priorities. The guiding principle is to address the most immediate threat to the patient, without losing sight of the competing considerations of the other phases.

During Resuscitation, reversing shock takes priority: clinicians must act promptly without overthinking the risks of iatrogenic fluid accumulation, while subsequent small boluses still permit repeated reassessment. Optimisation becomes increasingly important when initial interventions fail to restore perfusion, prompting detailed haemodynamic evaluation, including fluid responsiveness testing and the individualised decision of when to initiate vasopressors. Stabilisation does not abruptly end the Optimisation phase. Rather, it broadens the assessment to previously less prominent concerns, including contextual interpretation of fluid balance and fluid tolerance, sodium exposure, and maintenance fluid requirements. Regarding Evacuation, we agree that premature or excessive fluid removal may be harmful and would even argue that diuretics are overused. O'Connor et al. demonstrated that clinicians commonly report initiating fluid removal based on imperfect indicators (fluid balance and oedema), while citing increasing vasopressor requirements, rising lactate or signs of impaired tissue perfusion as reasons to reconsider—in other words, potentially only when patients are already drifting back towards shock. [3] Moreover, the overlooked observational follow-up of FACTT associated a conservative, diuretic-intensive fluid strategy with worse long-term cognitive outcomes, particularly executive dysfunction, despite improved short-term respiratory outcomes [4]. Although requiring confirmation, this finding challenges the assumption that more fluid removal is necessarily better. Such caution seems warranted given the modest benefits demonstrated so far: the recent ESICM guidelines on fluid removal report an increase in ICU-free days, but no statistically significant reduction in mortality [5]. VExUS may help identify venous congestion, but cannot alone establish whether de-resuscitation is appropriate or safe.

Throughout ROSE, physiological reasoning must prevail over rigid adherence to arbitrary rules: administering 30 mL/kg, routinely prescribing maintenance fluids—or, conversely, routinely withholding them—or starting diuretics to “zero” the fluid balance. Much of the controversy surrounding fluid therapy may reflect the inherent difficulty of translating heterogeneous bedside practice into clinical trials, and trial findings back to individual patients. Moreover, evolving insights and shifting standards of care may render yesterday's 'liberal' or 'restrictive' strategies difficult to interpret in today's context. Trials necessarily simplify this complexity into reproducible interventions; their findings should inform rather than replace bedside reasoning.

Ultimately, the art of intensive care medicine lies in integrating sometimes conflicting physiological signals with the imperfect generalisations of clinical trials to make the best possible decision despite inevitable uncertainty. Fluid stewardship is no exception, regardless of where the pendulum swings.

## Authors' contributions

M.V.E. and N.V.R. contributed to the concept of the review, drafted the manuscript and approved the final manuscript.

## Funding

Not applicable

## Ethics approval and consent to participate

Not applicable

## Competing interests

M.V.E has nothing to declare. N. V. R. received speaker’s fees from Baxter Healthcare and Nestlé Healthcare. N.V.R. is co-founder and executive board member of the International Fluid Academy (www.fluidacademy.org), a not-for-profit organization that receives unrestricted educational grants from the Industry.

Consent for publication

Not applicable

## Availability of data and material

Not applicable

## Declaration of Generative AI and AI-assisted technologies in the writing process

ChatGPT (OpenAI) was used solely to review the language of the manuscript. The authors reviewed and approved the final text and take full responsibility for its content.
